# Monthly Summary

**Published:** 1872-01

**Authors:** 


### MONTHLY SUMMARY.

---

*Chloral as an Antiseptic.*—Mr. Stodart, of Bristol, has recently examined the stomach, lung, heart, kidney, and spleen of a patient who died from an overdose of chloral hydrate. "The first thing," he says, "that struck me, was the very extraordinary way in which the several portions were preserved. Even now, although more than a week has elapsed since death, yet not the slightest sign of decomposition has taken place, nor any unpleasant odor. This doubtless is the effect of chloroform in the tissues."—*Med. Press and Circ.*

---

*Lemon for a Cough.*—Roast the lemon very carefully without burning it; when it is thoroughly hot, cut and squeeze into a cup upon three ounces of sugar, finely powdered. Take a spoonful whenever your cough troubles you. It is good and agreeable to the taste. Rarely has it been known to fail of giving relief.—*Druggists' Circular.*

---

*Treatment of Croup by Inhalation of Glycerine.*—A German physician, Dr. Stehverger, recommends the treatment of croup by the inhalation of pure glycerine through one or other of the well-known forms of atomizing apparatus. He was led to try this remedy for croup from observing its good effects in cases of hoarseness and loss of voice. After application the cough becomes more free and moist, and children are enabled to sleep almost immediately upon being relieved by the inhalation. It is, however, believed to be of importance to make use of the remedy early and frequently, as, if delayed, it may have no effect whatever. If the glycerine be pure, it may be used unmixed; if not, it should be diluted with a little water. The inhalations are repeated, according to the necessity of the case, at intervals of from half an hour to an hour and a half, and for about fifteen minutes at a time. The effect of the glycerine in this case is supposed to be due to the fact that the secretions of the mucuous membrane are thereby increased and tumefaction reduced.—*Druggists' Circular.*

---

*Influence of Tobacco in Diseases of Nerve-Centres.*—In the *Bulletin de l'Association France. "cont. l'Abus du Tabac,"* M. Tamisier states that out of fifty-nine grave affections of the nerve centres observed from 1860 to 1869 among men, forty occurred in smokers. In fifteen of hemiplegia, nine abused tobacco and two used it moderately ; four did not smoke. Of eighteen cases of paraplegia, five were great smokers, three moderate smokers, and ten abstained from tobacco. Out of twenty cases of locomotor ataxia, fourteen were great smokers, five moderate, and one abstainer. Tamisier thinks that it is especially, if not wholly to this cause, that we must attribute the disease in the majority of cases of hemiplegia and of ataxia he has noticed since 1860. M, Lefevre, of Louvain, thinks it indubitable that excessive smoking causes paralytic mania : because, 1st, nicotine causes in animals progressive enfeeblement of the muscles of motion up to paralysis, and congestion of the nerve-centres. 2nd, Analogous symtpoms have been noticed in numbers of persons who abused tobacco in smoking or chewing. 3d, It has been found in all countries that there is a constant relation between the consumption of tobacco and the increase of general paralysis.—*The Doctor. Boston Med. & Surg. Journal.*

---

*Transplantation of Mucous Membrane.*—At a meeting of the College of Physicians and Surgeons of Vienna, Dr. Czerny (*Am. Practitioner*) exhibited a case of transplantation of the mucous membrane of the nose upon a granulating surface of the upper arm. He spoke of three cases he had experimented on in this way, and one in which he had taken the mucous membrane from the mouth. The mucous membrane in one instance was taken from an extirpated polypus, and transplanted two hours after its removal. It had lost on its new ground its villi, and had changed into basement epithelium.—*N. Y. Med. Record.*

*To Avoid the Ague.*—The first suggestion, of course, is to leave those districts where this troublesome complaint prevails. Sometimes, however, one's residence cannot well be changed. To persons so circumstanced, there are preventions by the use of which the majority might generally escape it, which are referred to in the *Journal of Health* as follows:

1. Avoid exposing themselves to the malarial air after sunset and before sunrise. 2. Occupy rooms at night on the sunny side of the house and up stairs. 3. Build a fire in the house as soon as the dew begins to fall. The heat of the fire will do much to kill the malaria. 4. Keep the skin healthy and active by a thorough bath every day on rising, in a warm room, with sufficient friction to produce a healthy reaction. 5. Keep the bowels open by a proper diet. In nine cases out of ten the cause of ague would be easily overcome if the depurating organs were not overtaxed and morbid matters allowed to accumulate in the system to oppress it.

---

*An Enormous Collection of Earthy Matter in a Human Lung.*—By V. Gorup-Besanez.—The two cases were under the care of Prof. Zenker; V. Gorup-Besanez made the chemical examination of the lungs,

One was a woman who was employed in the manufacture of the books of thin red paper in which gold-leaf is placed. Fifty-seven grm. of one lung was incinerated, the ash treated with muriatic acid and examined for oxide of iron, of which 0.828 grm. were found. Supposing the oxide equally distributed through both lungs, the total amount would be between 21 and 22 grm.

The second case was a manufacturer of ultramarine. Two hundred and twenty seven grm. were incinerated and the ash remaining gave 3.1935 grm. siliceous sand, 0,3298 grm. quartz-sand, and 0.329 grm. of oxide ef iron.

Considering the total weight of both lungs 1500 grm., and these substances to be equally distributed, the total weight ef sand, &c., would be 29.86 grm.—*Annalen d. Chemie u. Pharmacie.*

*Posture of the Head in Sleeping.*—It is often a question among people who are unacquainted with anatomy and physiology, whether lying with head exalted or on a level with the body is the more unwholesome. Most, consulting their own case on this point, argue in favor of that which they prefer. Now, although many delight in bolstering up their heads at night and sleep soundly without injury, yet we declare it to be a dangerous habit. The vessels in which the blood passes from the heart to the head are always lessened in their cavities when the head is resting in bed higher than the body; therefore, in all diseases attended with fever the head should be pretty nearly on a level with the body; and people ought to accustom themselves to sleep thus and avoid danger.—*Home and Health.*

---

*Local Anæsthesia.*—Dr. Spessa (*Lancet*) "states in the *Bulletin des Sc. Med.* (Italy) that he has succeeded in preventing pain, during the slitting of a fistulous tract, by injecting a solution of morphine into the tract before the use of the knife. The same author had occasion to touch the vulva vegetation of a young girl with butter of antimony; the pain was very acute, but disappeared on the part being brushed over with a solution of morphia. A boy of fifteen, suffering from hip-joint disease, required an issue over and behind the great trochanter. An injection of morphine was first made over the region, and Vienna paste applied, which latter remained about eight minutes. The paste did not give any pain."—*Medical Cosmos.*

---

*Cause of Death from Chloroform.*—At a recent meeting of the Medical Library and Journal Association, Dr. A. H. Smith read an interesting paper on One of the Causes of Death from Chloloform, advancing the view that in some cases the serious results induced by this agent might be due to its local anæsthetic action upon the respiratory organs. Not only are the air-cells exposed directly to the vapor, but the blood brought to the lungs is loaded with the anæsthetic. The lecturer thought that the effect of this double dose might be to deaden the *besoin de respirer*, that instinctive sensation which—analogous to the feelings of hunger and thirst—impels the reflex act of respiration.—*Med. Gazette.*

*Zinc Poisoning.*—In the *Journal of Chemistry* we find reported a case of chronic zinc poisoning, which was traced to the use of a submerged galvanized iron pump. On inspecting the condition of the pump, it was found that the coating, over a portion of the surface, was entirely removed, and over the remainder was thoroughly corroded, so as to be readily removed with the finger. The prominent symptoms in this case were headache, nausea and gastric distress, pain in the larger joints, and partial paralysis of the lower limbs and the right arm. Recovery, even after removal of the cause, was very slow and imperfect.—*Detroit Review of Medicine.*

---

*Test for the Genuineness of Silver Plating.*—Van Dingler, in the *Polytechnische Journal*, gives the following simple method of distinguishing genuine silver from baser imitations:

Having carefully cleaned the metalic surface, place upon it a drop of a cold saturated solution of potassium bi-chromate in nitric acid, and wash off immediately with cold water. If the surface is silver a blood-red stain, consisting of silver chromate is formed. On German silver or Britannia ware a black or brown spot results.—*New Remedies.*

---

*Test for Arsenic.*—Bethendorff has lately discovered an extremely delicate test for arsenic, which is, moreover, unaffected by the presence of antimony. The suspected liquid is mixed with hydrochloric acid until fumes are apparent, when chloride of tin is added, which produces a basic precipitate containing the greater part of the arsenic as a metal, mixed with oxide of tin. It is stated that arsenic may be detected by this test when it forms one millioneth part only of the solution.—*Detroit Review of Medicine.*

---

*Soda Mint.*—This is often prescribed as an antacid and carminative, and is prepared, according to the "Journal of Pharmacy," as follows:

℞.—Sodæ Bicarb, ℥ss.;

Spt. Ammon. Aromat., ʒi.;

Aquæ Menthæ Pip., Oj.

M. Dose, from a desert spoonful to a tablespoonful for adults; from half to one teaspoonful for infants.

*Sulpho Urea.*—Dr. B. W. Richardson, of London, has been recently experimenting with this anæsthetic. It is a light, volatile fluid, having an agreeable vapor, and in some cases insensibility was produced by it in fifteen minutes.— *Virginia Clinical Record.*

---

*Bullet over Nineteen Years in the Brain.*—The *Journal of Psychological Medicine*, July, 1871, contains the following, which is taken originally from an Itallian Journal : Joseph Solen, a lawyer in Venice, was shot in a duel, June 16th, 1850. The ball entered the head above the right ear. Prof. Cortesi saw the wound five hours afterwards. For a considerable time his memory was weak, and vision was somewhat disturbed. In time, however, these two disturbances disappeared, his mind became as clear and his judgment as good as ever. He now complained of nothing but a painful sensation in the back and in the lower extremities, especially in the right, which became more acute wnen he coughed or sneezed, and extended to the hcad. He died of pneumonia in December, 1869. The autopsy displayed a funnel-shaped cicatrix above the right ear, and a notable thickening of the skull. On the petrous portion of the temporal bone, and on the border of the tentorium, a dark body was found which consisted of two pieces sf lead separated from each other by a splinter of bone. Running into the substance of the brain there was a canal ten centimetres in length at the bottom of which was found a piece of bone.—*Medical Times.*

---

*Test Solutien for Pus.*—A saturated alcoholic solution of guaiacum exposed to the air till it acqnires the property of giving a green color with potassium iodide, is said by Dr. Day, of Geelong, to be a good test for pus. When added to liquid pus, or dried pus moistened, it gives a clear blue color.—*Michigan Medical Journal.*

---

*Chloral as an Antiseptic.*—Mr. Stodart, of Bristol, had occasion, recently, to examine the viscera of a patient who had died from an over-dose of chloral. He observed that the tissues were preserved in an extraordinary manner. Even after the lapse of a week, no sign of decomposition was apparent.—*Medical Press and Cirular.*

